# CVN293, an Investigational Inhibitor of KCNK13 Targeting NLRP3‐Mediated Neuroinflammation in Neurodegenerative Disease: A Phase 1 Study to Investigate the Safety, Tolerability, and Pharmacokinetics.

**DOI:** 10.1002/alz70859_106386

**Published:** 2025-12-25

**Authors:** Louise Dickson, Roberto Tolando, Celina Scholl, Michelle Charles, Dino Ossola, Kim Matthews, Graham Scott, Martin Bexon, Jasminne Castricum, Philip Kremer, Craig Thompson, Sagar Vaidaya, Lee A Dawson, Mark Carlton, Nicola Brice

**Affiliations:** ^1^ Cerevance Ltd, Cambridge, Cambridgeshire United Kingdom; ^2^ Cerevance, San Diego USA; ^3^ GS Drug Development Sciences, East Grinstead, West Sussex United Kingdom; ^4^ Bexon Clinical Consulting, Upper Montclair USA; ^5^ Centre for Human Drug Research, Leiden Netherlands; ^6^ Cerevance, San Diego, CA USA

## Abstract

**Background:**

CVN293 is a novel, investigational, small molecule inhibitor of the two‐pore domain potassium channel KCNK13. KCNK13 is selectively expressed in microglia and mediates the potassium efflux required for activation of the NLRP3 inflammasome complex. NLRP3‐mediated inflammation has been identified as a central mediator of neuroinflammation in neurodegenerative diseases. Through inhibition of KCNK13 and microglial proinflammatory processes, CVN293 has the potential to dampen neuroinflammation across neurodegenerative diseases.

**Method:**

This Phase 1 single/multiple ascending dose (SAD/MAD) study investigated the safety, tolerability and pharmacokinetics of orally administered CVN293 in healthy subjects. Following a randomized, double‐blind and placebo‐controlled design, subjects received either CVN293 or placebo with SAD cohorts receiving single doses up to 1000mg (all fasted; 150mg was also tested under fed conditions). MAD cohorts received repeated doses over 14 days, up to 750mg (375mg twice daily). CSF sampling was conducted in both SAD and MAD cohorts.

**Result:**

CVN293 was generally well‐tolerated following single and 14‐day multiple dosing and all subjects completed the treatment period. There were no severe or dose‐limiting adverse events (AEs), treatment‐related discontinuations, or clinically meaningful changes in vital signs or laboratory parameters. All related AEs were considered mild. In both the SAD and MAD phases, CVN293 plasma exposure increased in a generally dose proportional manner. Penetrance of CVN293 into the cerebrospinal fluid (CSF) was demonstrated in both the SAD (150mg) and MAD (150mg, 75mg twice daily) studies.

**Conclusion:**

This Phase 1 study demonstrated a favourable tolerability profile for CVN293 in healthy adults and supports the continued development of this novel KCNK13 inhibitor for neurodegenerative diseases driven by neuroinflammation.

